# Development and evaluation of a next-generation sequencing methodology for measles virus using Oxford Nanopore Technology

**DOI:** 10.1128/jcm.01456-24

**Published:** 2025-02-04

**Authors:** Vanessa Zubach, Gurasis Osahan, Kurt Kolsun, Claudia Pfeifer, Alberto Severini, Joanne Hiebert

**Affiliations:** 1 Measles, mumps and rubella Unit, National Microbiology Laboratory, Public Health Agency of Canada, JC Wilt infectious Diseases Research Centre 85072 , Winnipeg, Manitoba, Canada; 2 Department of Medical Microbiology and Infectious Diseases, Faculty of Health Sciences, University of Manitoba 574854 , Winnipeg, Manitoba, Canada; The University of North Carolina at Chapel Hill School of Medicine, Chapel Hill, North Carolina, USA

**Keywords:** measles, Nanopore, whole genome sequence

## Abstract

We report the development of a bench protocol and evaluation of bioinformatics pipelines for the whole genome sequence (WGS) of measles virus (MeV) genotype D8. We established a bench protocol using 1 kb amplicons tiling the MeV WGS. Four different pipeline parameters were assessed based on two basecallers and two quality thresholds: Guppy simplex with Q-score thresholds of 20 and 25 (G20 and G25), and Dorado duplex with Q-score thresholds of 20 and 25 (D20 and D25). Using a reference genome, we determined that complete genomes were obtained down to 10 copies/µL with all four parameters; however, errors began to be detected in the consensus sequence at 100 copies/µL. A panel of specimens from 32 measles cases, for which measles WGS had been obtained by other methods (reference sequences), was used to assess the utility and accuracy of the Oxford Nanopore Technologies (ONT) for the purposes of measles surveillance. We found that a crossing point (Cp) value of 31 (corresponding to approximately 100 copies/µL) or less could be considered a predictor for the generation of accurate and complete WGS. The GQ20 parameter achieved the most complete genomes (75%) and had the most identical sequences (84.4%). Error rates compared with the reference sequences for all parameters were below one nucleotide per whole genome. After assessing the reproducibility, GQ20 had the most identical sequences (97.4%). Finally, we inserted ONT-generated WGS and reference sequences into outbreaks with known epidemiological links, and our results show that the ONT WGS matches the epidemiological data. This evaluation establishes that NGS generated by ONT produces accurate and reliable MeV WGS.

**IMPORTANCE:**

The use of ONT-sequencing platforms has the potential to expand the availability of measles sequencing as a result of its relatively lower cost and portability. This study establishes that measles sequences generated by ONT are accurate and reliable. This will enable sequencing in global regions where there is a lack of sequence data (which also tend to be the measles exporting regions) and more timely sequencing in low incidence settings, due also to the lower number of samples needed for the ONT platform. More timely generation of these data enables better investigation of cases, which informs public health response and outbreak management in measles-eliminated countries.

## INTRODUCTION

Infection with the highly contagious measles virus (MeV) is preventable with the live-attenuated vaccine that was developed in the 1960s (1). During 2020–2022 due to the COVID-19 pandemic, mass vaccination campaigns globally were canceled or postponed, resulting in reduced vaccination coverage worldwide (2). The global MeV vaccine coverage rate of the first dose is 83% and for the second dose is 74%, both remaining well under the 95% coverage with two doses necessary to protect communities from outbreaks (3). In 2024, several countries across the world have reported spikes in cases of measles compared with 2023 (4–7). In North America where vaccination rates are high, most measles cases occur in unvaccinated travelers (8).

Genetic characterization of wild-type MeV is a critical component of the global measles surveillance program (9). Sequence data can be used to map the transmission of MeV by allowing linkages between cases and/or outbreaks and revealing possible sources of importation (10). The World Health Organization (WHO) recommends molecular surveillance of every measles outbreak by genotyping the 450 nucleotide (nt) region of the C-terminus end of the nucleoprotein (N) gene (N450) (11). Historically, there were 24 MeV genotypes circulating, but since 2021, only two genotypes (D8 and B3) remain active (9). Due to this decreasing genetic diversity, genotyping of the N450 is not always adequate for monitoring transmission, and a higher resolution of sequencing is needed to resolve outbreaks (12).

Sanger sequencing developed in the 1970s remains the gold standard for determination of the nucleic acid sequence (13). However, Sanger sequencing is not ideal in high-throughput situations because it requires a separate reaction for each fragment, requires a higher input of DNA, and only supports sequences up to 1,000 bp (13). Next-generation sequencing (NGS) has been evolving over the last 15 years with Illumina technology widely used because it produces high-quality reads (14). The high cost of the equipment and materials, and the often-time-consuming library preparations make real-time whole genome sequence (WGS) outbreak analysis with Illumina technology challenging and not suitable for all settings. Oxford Nanopore Technologies (ONT) sequencing products have been around since 2014, but due to their high error rate, they have not been widely used (15). A low error rate is particularly important for measles molecular surveillance, since just a two or three nucleotide difference over the 15 kb genome is sometimes enough to differentiate unrelated outbreaks (16). Over the years, ONT error rates have improved dramatically with changes in sequencing methodology, the software for base detection, and additional library preparation modifications (17). The newest R10 flow cells have pores with two sensing regions to aid in accuracy, especially in homopolymer regions (18). ONT claims with their newest flow cells (R10.4.1) and version 14 Kit chemistry, a raw read accuracy of 99% and above can be achieved (18). This has been demonstrated to overcome some of the known challenges in homopolymer regions that often result in insertions or deletions (19). Basecalling decodes the electrical current signal-to-nucleotide sequence; it is critical for data accuracy, and ONT has also been making advances with its technology (20). Basecalling can be done in simplex by Guppy or duplex by Dorado. Simplex data are generated when a single strand of DNA is sequenced, and duplex data are generated when the complement strand is read immediately after the template strand, and the consensus basecall for both strands leads to a further increase in accuracy (18). ONT offers the flexibility of running small batches with a Flongle Flow cell or very large batches on the PromethION. The lower cost, compact size, flexibility to process varying numbers of samples, ultra-long read lengths, and real-time sequencing capabilities make ONT a financially attractive option for many laboratories to perform real-time WGS of cases (18). Scientific reports comparing ONT and Illumina WGS for MeV are lacking, but there have been reports comparing ONT and Illumina WGS for other pathogens, such as SARS-CoV-2, hepatitis B virus, and gram-negative bacteria (21–23).

Using ONT latest technologies, R10.4.1 flow cells with version 14 kit chemistry, and two recent basecallers (simplex Guppy V6.5.7 and duplex Dorado V0.4.1), we sought to develop an ONT bench protocol and evaluate bioinformatics pipelines for the WGS of MeV genotype D8 specimens. Two basecallers were evaluated, each applied with two distinct quality thresholds (Q20 and Q25), resulting in a total of four ONT parameter combinations. We established robust, data-driven minimum depth of coverage thresholds and compared the ONT-generated WGS using surveillance specimens from 32 measles cases to previously characterized WGS generated by Illumina or combined Illumina and Sanger, which we denote as reference sequences (16). Additionally, WGS were generated by ONT from four additional specimens, and their accuracy was validated by the available epidemiological information.

## MATERIALS AND METHODS

### Design of MeV-tiled primers for ONT libraries

Initially, two different sets of primers were designed, one approach was to amplify 400 bp regions, and the other approach was to amplify 1 kb regions covering the entire measles D8 genome using Primal Scheme (24) against a reference sequence for MeV genotype D8 (MH356245) (Fig. S1 and S2). Both approaches with amplicons of 400 bp or 1 kb length were assessed, as was the use of water or betaine at the primer annealing step of the reverse transcription reaction. Supplemental primers were required in the matrix to fusion non-coding region (MF-NCR) and at the ends of the genome (inhouse and Primer3 software (25) designed) to obtain complete coverage over the whole genome, minus the combined 216 nt long termini. WGS was accomplished with the 1 kb amplicons, supplemental primers, and by splitting all primers into six separate pools, ensuring neighboring amplicons did not overlap within the same pool. Fig. S3 represents the final choice of primers and their pools. Primer sequences, pools, and concentrations are provided in Table 1. These primers were checked against measles genotypes B3, D4 D8, and H1 using Primer-BLAST (26), allowing up to two mismatches which resulted in 228 database matches, 86 of which were genotype D8.

**TABLE 1:** Measles tiling primers used in ONT library preparation showing the primer pool, name, sequence, final concentration, and design source

Primer pool	Name	Sequence	Final concentration [μM]	Design source
1	D8_165–1113-F	AGAAACAAGGACAAACCACCCA	0.25	Primal scheme
1	D8_165–1113-R	AGTTCCACTCCTACTCCCATGG	0.25	Primal scheme
1	D8_1865–2853-F	GAATGCATCCGGGCTCTCAA	0.5	Primal scheme
1	D8_1865–2853-R	TTTTGCCTGTTGATCTGCTTCTT	0.5	Primal scheme
1	D8_3490–4456-F	CATGGGACATCAAAGGGTCGAT	0.25	Primal scheme
1	D8_3490–4456-R	GGTCGTTTTTGGGCATTGCTG	0.25	Primal scheme
1	D8_6886–7804-F	TTGGAGGATGCCAAGGAATTGT	0.25	Primal scheme
1	D8_6886–7804-R	CCCTTTGAGACAGCTAGGAACTG	0.25	Primal scheme
1	D8_8603–9533-F	GGCTCAGGGATGGACCTATACA	0.5	Primal scheme
1	D8_8603–9533-R	CACGGATCTTCCTCGTTGACTC	0.5	Primal scheme
1	D8_10278–11234-F	TGACATACATCTGACAGGGGAGA	0.5	Primal scheme
1	D8_10278–11234-R	TGGTCTCATATCTCCAATTAAGGCA	0.5	Primal scheme
1	D8_11999–12956-F	TGTAGTCATACCCCTCCTCACA	0.25	Primal scheme
1	D8_11999–12956-R	AAGCCCATGAGTACACTGTTGC	0.25	Primal scheme
1	D8_13729–14673-F	GCCATCAATTGGGCATTTGATGT	0.25	Primal scheme
1	D8_13729–14673-R	TGACCAGATCTAGAATTGGCGG	0.25	Primal scheme
1	D8_14783–15750-F	TGTCAAGGTGCTCTTTAACGGG	0.25	Primal scheme
1	D8_14783–15750-R	AGGGCACTGTATCCGACTAACT	0.25	Primal scheme
2	D8_1038–1975-F	CAGCAAATGGGAGAAACTGCAC	0.25	Primal scheme
2	D8_1038–1975-R	ATGGTTTGCTGAGACCCGAAC	0.25	Primal scheme
2	D8_2752–3713-F	GATGAGCTGTTCTCCGATGTCC	0.5	Primal scheme
2	D8_2752–3713-R	CTATGTCAAGCTCAGTGGCCTC	0.5	Primal scheme
2	D8_6004–6952-F	CAGGGTGTCCAAGACTACATCA	0.25	Primal scheme
2	D8_6004–6952-R	CCTCCAAGACACACTGCAATCA	0.25	Primal scheme
2	D8_7711–8672-F	CAACCCGCCAGAGAGAATCAAA	0.25	Primal scheme
2	D8_7711–8672-R	CTCGGTATCCACTCCAATGTGT	0.25	Primal scheme
2	D8_9450–10382-F	TGTGGAAGTTGGGAATGTCATCA	0.25	Primal scheme
2	D8_9450–10382-R	TGGCATGACCTTTCATCAGAGT	0.25	Primal scheme
2	D8_11109–12079-F	CACACAAGTACCAGGAACGTGA	0.25	Primal scheme
2	D8_11109–12079-R	TGGATCACCGATGTTTCTGACA	0.25	Primal scheme
2	D8_12865–13820-F	GGAAACATCATCCTTGAGAGTCCC	0.25	Primal scheme
2	D8_12865–13820-R	TTGGGTGGCTTAGAGCATTGAC	0.25	Primal scheme
2	D8_14597–15524-F	GAAGACGGCTTGTTCTTGGGT	0.25	Primal scheme
2	D8_14597–15524-R	CCCAAAATTTGCGGGTGATCCT	0.25	Primal scheme
3	D8_118–478-F	GATTAGAGACATCCGAAATGGCCACACT	0.25	Primer 3 software
3	D8_118–478-R	CATCATGTGAAAAGTATTGGTCCGCCTCATCC	0.25	Primer 3 software
3	D8_5716–6057-F	GGAGACTACTGAGAACAGTTCTGGAACCAA	0.25	In house
3	D8_5716–6057-R	ATAGTATCTGAGCAATTTGAGCCCTAGCTT	0.25	In house
4	D8_4355–4860-F	CAGATGCAAGATAGTAAGAATCCAG	0.25	In house
4	D8_4355–4860-R	CTTGGCCCTTAGTTTTGTTTAG	0.25	In house
5	D8_25–1025-F	CCAAACAAAGTTGGGTAAGGATAG	0.25	Primer 3 software
5	D8_25–1025-R	CCATRTARGGTGCAGTTTC	0.25	Primer 3 software
5	D8_4812–5577-F	CACAAGCGACCGAGGTGAC	0.25	In house
5	D8_4812–5577-R	CGAGTCATAACTTTGTAGCTTGC	0.25	In house
6	D8_5432–5813-F	CCTAAAGGAGACACCGAGAATCCCAG	0.25	In house
6	D8_5432–5813-R	CAACGCCTAGGGCCGCACCT	0.25	In house
6	D8_14854–15865-F	GTYAGTAATATCCCYACCTCTAG	0.25	Primer 3 software
6	D8_14854–15865-R	CAGACAAAGCTGGGAATAGAAACTTCG	0.25	Primer 3 software

### Measles specimens, nucleic acid extraction, and RT-PCR

MeV RT-PCR-positive specimens (*n* = 48) that were collected from confirmed cases of measles (genotype D8) occurring in Canada between 2018 and 2023 and were referred to the Public Health Agency of Canada’s (PHAC) National Microbiology Laboratory (NML) for measles genotype surveillance were included. Research ethics board review was not required as per Article 2.5 of the Tri-Council Policy Statement: Ethical Conduct for Research Involving Humans – TCPS 2 (2022) (27). The types of samples in decreasing frequency were nasopharyngeal swabs, urine, throat swabs, and nasopharyngeal aspirates in viral transport media. Previously generated WGS (termed reference sequences) were available for 44 specimens, whereas another four specimens did not have a reference WGS. These four specimens had epidemiologically linked data available for comparison.

Total nucleic acids were extracted from 200 µL of specimen or 280 µL of measles viral isolate using the MagNA Pure 96 DNA and Viral NA large volume kit on the MagNA pure 96 (Roche Diagnostics Corp., Indianapolis, IN, USA) or with the QIAamp Viral RNA Mini Kit (QIAGEN) and eluted in 50 µL, following manufacturer’s instructions. Extracted RNA was stored at −80℃ if not used immediately. Real-time RT-PCR was performed on a Roche LightCycler 480 to obtain a crossing point (Cp) value for these samples, targeting the nucleoprotein gene as previously described (28).

#### Measles reference genome

A genotype D8 measles virus isolate (VR-1980) with downloadable genome sequence data was purchased from American Type Culture Collection (ATCC). The measles reference genome nucleic acid extract was quantified with a QuantStudio Absolute Q Digital PCR (dPCR) system (Applied Biosystems) using the nucleoprotein gene RT-PCR primers. The extract was diluted to 10^6^ copies/µL and a 10-fold dilution series, to 1 copy/µL, was prepared in a 10 mM Tris-HCL solution with 5 µg/mL Yeast tRNA (ThermoFisher Scientific AM9855G, AM7119).

#### Validation samples and reference sequences

WGS from 32 genotype D8 clinical specimens that were previously generated using Illumina or a combination of Illumina and Sanger (typically the MF-NCR) were used as reference sequences for the purposes of this evaluation and used to validate the ONT-generated WGS (16). The sequences were 15,678 nucleotides in length. Majority reads (>50%) were used for consensus calling. For the Illumina/Sanger data, whenever the coverage depth of 10 was not met, the sequence was patched with Sanger-generated sequence Details about the samples and the sequence generation are provided in reference 16.

#### Oxford nanopore library preparation

Reverse-transcription was performed with 7.5 µL of extracted total nucleic acid using Superscript IV (ThermoFisher Scientific catalog 18090200) according to the manufacturer’s instructions but included betaine (Sigma-Aldrich catalog B0300) to a final concentration of 0.2 M in a 30 µL reaction. Multiplex PCR occurred when 2 µL of the prepared cDNA was amplified in six different pools using Q5 HotStart High Fidelity 2× mastermix (New England BioLabs catalog M0494). Multiplex PCR reactions were carried out in 12.5 µL according to the manufacturer’s guidelines. Initial denaturation took place at 98°C for 30 s, amplification followed with 34 cycles of denaturation at 98°C for 15 s, and primer annealing/extension at 68°C for 5 min. After PCR amplification, DNA from the six pools were combined and purified using AMPure XP beads (Beckman Coulter catalog A63882, 0.75X bead ratio), then quantified on the Qubit (ThermoFisher) using the dsDNA, broad range kit (ThermoFisher catalog Q33265). Two hundred femtomoles (200 fmol) of each sample were further prepared using the ONT Native Barcoding kit (ONT catalog #SQK-NBD114.24) according to the manufacturers' instructions with the addition of a purification step with AMPure XP beads (0.4× bead ratio) after the barcode ligation. This additional bead cleanup was performed to reduce the risk of barcodes ligating when samples were pooled, particularly in cases where the EDTA added might not have been sufficient to completely stop the enzymatic reaction. The library preparation was quantified on Qubit using a dsDNA High Sensitivity kit (ThermoFisher catalog #Q32850), and 10–20 fmol of the library was loaded onto the flow cell. Up to 24 samples, including at least one no-template control and positive control were multiplexed on a R10.4.1 flow cell (ONT catalog #FLO-MIN114) and sequenced on a MinION MK1C device. Library preparation required 2 days, and after 48 h, the run was terminated.

### Bioinformatics pipeline

The resulting raw signal data in HDF5 files (FAST5 or POD5) were basecalled and demultiplexed using Guppy simplex (V6.5.7) and Dorado duplex (V0.4.1) using the Super High Accuracy model (V4.1.0). Resulting demultiplexed FASTQ reads were processed using the NanoGO (0.3.0) pipeline (https://anaconda.org/gosahan/nanogo). Briefly, NanoGO is made up of 3 stages: Preprocessing, Data Analysis, and Quality Control. The preprocessing stage includes Porechop (v0.3.2) to remove adapter and barcode sequences, followed by Cutadapt (v4.5) to remove primer sequences, low-quality reads, and low-quality nucleotides. Importantly, Cutadapt was also used to generate the data set employed for testing different Q-score parameters. The data analysis stage includes BCFTools (v1.11), Mafft (v7.520), Medaka (v1.8.0), Minimap2 (v2.26), QuasiGO (v0.1.6), Samtools (1.11), and iVAR (1.4.2). Medaka, QuasiGO, and iVAR each generate a consensus sequence, which are then aligned using Mafft, with the majority consensus used to produce the final consensus sequence. Initially, two different minimum quality Phred thresholds were used for data analysis, a minimum quality of 20 and 25. Finally, the Quality Control stage employs FastQC (v0.12.1), Kraken2 (v2.1.3), MultiQC (v1.18), NanoStat (v1.6.0), and QualiMap (v2.3) to assess quality control parameters.

#### Down-sampling and minimum depth of coverage data

Picard Downsample Sam (v3.3.0) was used to randomly downsample reads using the BAM files obtained by sequencing the ATCC VR-1980 reference genome (10^6^ copies/µL) in triplicate. Eleven 2-fold downsample intervals were used (specific proportions were 50, 25, 12.5, 6.25, 3.13, 1.56, 0.78, 0.39, 0.19, 0.09, and 0.05). QuasiGO (v.0.2.0) was used to process and analyze the reads.

In addition, ONT read data obtained from genotype D8 viral isolates (*n* = 4), sequenced at different dilutions and surveillance specimens (*n* = 48), were used as a data set to analyze and establish the minimum depth of coverage thresholds. Whole genome sequences were previously obtained as described for the reference sequences. Some of the samples had been sequenced multiple times as part of the development of the library protocol, and as a result, there were sequence data available from 201 templates basecalled by Guppy (G), and 123 templates basecalled by Dorado (D), where only POD5 read data could be used. The QuasiGO outputs the depth (or count) of reads by individual nucleotide, or gaps in the read, for each genome position in a spreadsheet format. Using the matched reference sequence as a comparator, these read counts were binned as correct or incorrect for each nucleotide position. This output was also used to explore the rates of errors and deletions in the read pile-up (the sub-consensus).

### Data analysis

Statistical analysis was computed in GraphPad Prism (v10.1.2) and Microsoft Excel (2016). Alignments for accuracy, reproducibility, and BAM analysis of Illumina generated sequences were done in Geneious Prime (v2023.2.1). Phylogenetic analysis was performed using Maximum Likelihood trees generated in Mega X (v2023.2.1) (29) using 500 bootstrap replicates. Interactive Tree of Life (iTOL)(30) was used for tree visualization enhancements. Plots and charts were generated in BioRender (https://app.biorender.com/), R Studio (2023.12.1), Microsoft Excel (2016) and GraphPad Prism (v10.1.2).

## RESULTS

### Amplicon tiling design

Two tiling schemes/approaches of different amplicon sizes, 400 bp and 1 kb, were assessed, each in two reaction pools (Fig. S1 and S2), with and without the addition of 0.2 M betaine to the reverse transcription reactions. The 1 kb amplicon tiling scheme resulted in higher coverage depth (Fig. S4). The larger amplicon size was also attractive in that fewer primers were needed. The addition of betaine with both tiling schemes improved the consistency of coverage and reduced gaps in the consensus sequences (Fig. S4). However, there was a significant drop out in certain regions, especially in the non-coding region between the matrix and fusion genes (MF-NCR) (Fig. S4). Supplemental primers were added to the 1 kb tiling scheme, and select primer concentrations were increased to a final concentration of 0.5 µM to optimize coverage across the genome. The final amplicon tiling scheme consisted of six primer pools and the use of betaine (Table 1; Fig. S3).

### Establishment of a minimum depth of coverage thresholds

**Fig 1:**
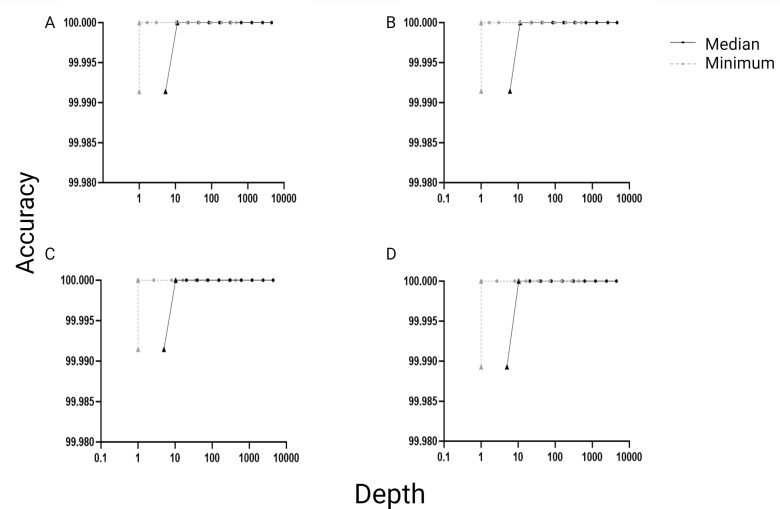
Assessment of consensus sequence accuracy against the minimum (gray-dashed lines) and median depths (black solid lines) on downsampled data from reference genome VR-1980. ONT sequence data were downsampled by 50% 11 times. Accuracy was calculated at each downsampling interval and plotted against the median and minimum depths for each of the four parameters. (A) Guppy Q20, (B) Dorado Q20, (C) Guppy Q25, and (D) Dorado Q25. The sequencing was done in triplicate, and the triplicates were downsampled. Average values of the downsampled triplicates were used. Minimum depth values of zero were excluded so that they could be plotted on a logarithmic scale. Triangles denote incomplete genomes.

A measles genotype D8 reference genome was obtained from ATCC (VR-1980), sequenced in triplicate, and the data were downsampled in 50% intervals 11 times. The consensus genome sequences were 100% accurate in comparison to the sequence provided by ATCC when basecalled by Guppy (G) or Dorado (D) using either minimum quality Phred scores of 20 (Q20) or 25 (Q25). The consensus sequences remained accurate until the second last interval with median coverage depths of 10–11 reads and minimum depths of 1 (Fig. 1). The final interval resulted in median depths of 5–6 reads; full-length genomes were not obtained with any basecalling parameter, and the consensus sequences were no longer accurate.

The reference genome used for downsampling was a cultured measles virus and would be expected to be a relatively clean starting template. A sequencing protocol for surveillance purposes would ideally use clinical specimens as starting material, which contains relatively lower amounts of on-target sequence in a more diverse genetic mixture containing other microbial organisms in addition to the host material. Thus, we analyzed the read data derived from the clinically obtained specimens (*n* = 48) as well as dilutions of four viral isolates. Similarly to the reference genome, consensus sequences were mostly accurate across the genome, in comparison to their established reference WGS, providing little data with which to inform minimum coverage depths for clinical specimens. Although downsampling is a commonly used approach, it randomly removes data irrespective of coverage and accuracy, which could result in the loss of inaccurate read data.

**Fig 2:**
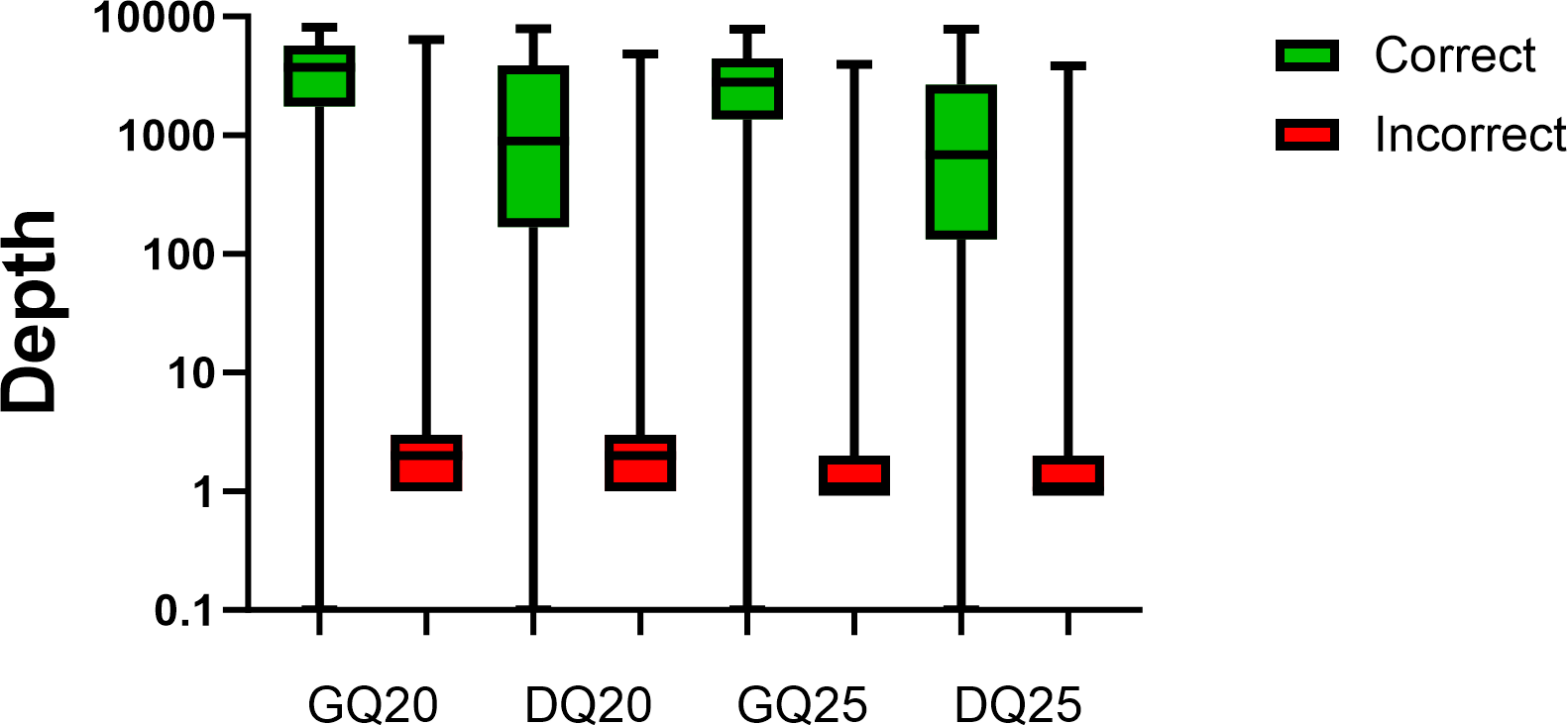
Summary of subconsensus read depths at each position in the genome binned as correct (green) or incorrect (red) using four parameters. GQ20: Guppy basecaller, quality Phred score 20, DQ20: Dorado basecaller, quality Phred score 20, GQ25: Guppy basecaller, quality Phred score 25, DQ25: Dorado basecaller, and quality Phred score 25. The whiskers represent minimum and maximum read depths, the boxes capture 25%–75% of the read depth values, and the median is plotted as a line in the boxes. Positions with no incorrect reads (zeros) were omitted. The Y axis is in the logarithmic scale.

As another approach, we mined the read pile-up data at each nucleotide position, quantified in the QuasiGO output, to explicitly explore the inaccurate reads and utilize them to establish minimum coverage cutoffs. For this purpose, all the reads for each position were binned as correct (matching the established reference WGS) or incorrect (not matching the established reference WGS), and the data were summarized (Fig. 2). Most positions had no more than 1–3 reads that were binned as incorrect (interquartile ranges were GQ20: 1–3, DQ20: 1–3, GQ25: 1–2, and DQ25: 1–2), whereas the number of correct reads were in the hundreds or more (Fig. 2). There were some sequence errors at the consensus level, which were reflected in read depths in the thousands for the data binned as incorrect (Fig. 2). The 95th percentile depths of read data binned as incorrect were ten for GQ20, ten for DQ20, eight for GQ25, and six for DQ25 and were likely skewed by the read depths for consensus level errors. Nonetheless, the 95th percentiles were selected as the minimum depth of coverage requirement as a conservative approach with minimal impact on overall coverage (completion of genomes). These depth cutoffs were also consistent with the downsampling analysis, and the cutoff of 10 which has been commonly adopted for measles WGS obtained with Illumina platforms.

### Reference genome dilutions: completion, accuracy, and reproducibility

One million (10^6^) copies/µL of reference genome VR-1980 were serially diluted in 10-fold increments to 1 copy/µL, sequenced in triplicate, and analyzed with all four parameters (Table 2). Down to 10 copies/µL, all four parameters were able to obtain complete coverage of the genome, with the minimum depth of coverage requirement applied. At 1 copy/µL, DQ25 obtained two of three replicates complete with the appropriate depth of coverage, whereas the other parameters did not yield any complete genomes. Consensus genome sequences were 100% accurate for all parameters down to 1,000 copies/µL, with the exception of 1 error in one replicate for all parameters at 10^4^ copies/µL. Sequencing errors began to accumulate at 100 copies/µL, which corresponds to a Cp value of 30.58. Using this same dilution series tested in triplicate, we calculated the reproducibility of the entire method, with an overall reproducibility of 99.99% for all four parameters (Table 2).

**TABLE 2:** Outcome of sequencing a 10-fold dilution series of a measles genotype D8 reference genome (ATCC VR-1980). Each dilution was sequenced in triplicate

	10^6^ copies/µL	10^5^ copies/µL	10^4^ copies/µL	10^3^ copies/µL	10^2^ copies/µL	10 Copies/µL	1 Copy/µL
CP value	17.95	21.38	24.89	27.74	30.58	34.04	36.95
Median depth (min–max)							
Guppy Q20	5293–5899	5594–6327	6125–6304	5311–5548	5270–5509	762–4804	29–94
Dorado Q20	5322–5891	5620–6173	6279–6445	5333–5566	5300–5571	690–4770	29–107
Guppy Q25	5108–5802	5360–6224	6081–6278	5246–5531	5003–5622	728–4449	26–85
Dorado Q25	5195–5867	5440–6196	6181–6341	5287–5558	5068–5690	704–4438	26–97
No. of complete genomes							
Guppy Q20	3	3	3	3	3	3	0
Dorado Q20	3	3	3	3	3	3	0
Guppy Q25	3	3	3	3	3	3	0
Dorado Q25	3	3	3	3	3	3	2
No. of mismatches in complete genomes (min–max)							
Guppy Q20	0 (0–0)	0 (0–0)	0.3 (0–1)	0 (0–0)	1.3 (0–2)	2.7 (1–4)	
Dorado Q20	0 (0–0)	0 (0–0)	0.3 (0–1)	0 (0–0)	1.3 (0–2)	3 (2–4)	
Guppy Q25	0 (0–0)	0 (0–0)	0.3 (0–1)	0 (0–0)	1 (0–2)	2.7 (1–4)	
Dorado Q25	0 (0–0)	0 (0–0)	0.3 (0–1)	0 (0–0)	1 (0–2)	3 (2–4)	1.5 (1, 2)
Reproducibility of complete genomes, %							
Guppy Q20	100	100	99.997	100	99.987	99.980	
Dorado Q20	100	100	99.997	100	99.987	99.977	
Guppy Q25	100	100	99.997	100	99.990	99.980	
Dorado Q25	100	100	99.997	100	99.990	99.980	99.985

### WGS sequencing with surveillance specimens

**Fig 3:**
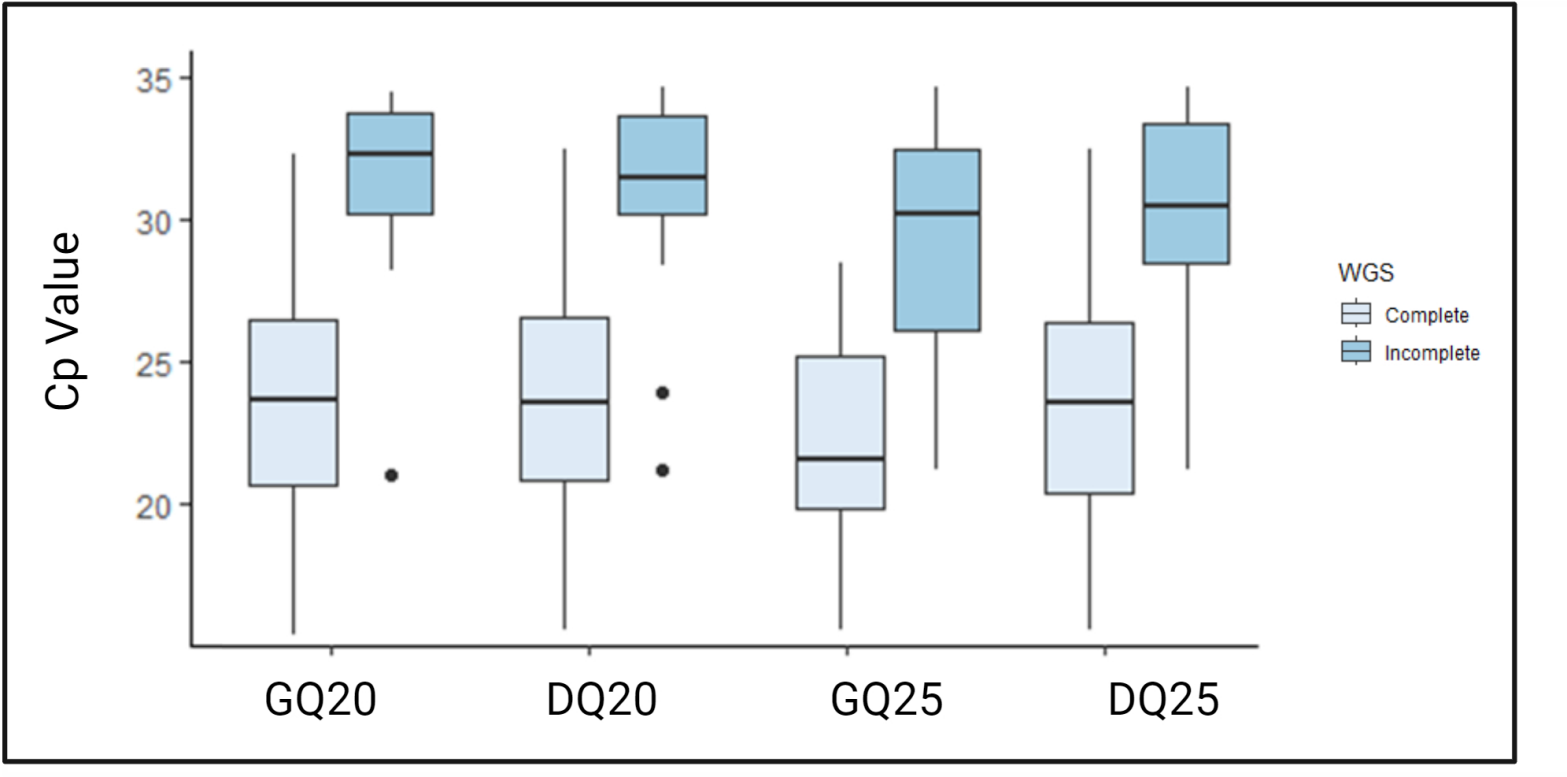
Distribution of measles real-time RT-PCR Cp values for 48 clinical specimens, grouped in pairs by the four pipeline parameters as complete (left in the pairs, lighter shade) and incomplete (right in the pairs, darker shade). There was a significant difference between the Cp values of complete and incomplete (Mann Whitney) with *P* values < 0.0001 for all pairs. GQ20: Guppy basecaller, quality Phred score 20, DQ20: Dorado basecaller, quality Phred score 20, GQ25: Guppy basecaller, quality Phred score 25, DQ25: Dorado basecaller, quality Phred score 25. Note the Roche Lightcycler does not provide values for the last five cycles; these results (>35) were set to 35 for the purpose of this analysis.

In total, WGS was attempted from 48 surveillance specimens. Using the established depth of coverage cutoffs and the four pipeline parameters, complete genomes, defined as complete read coverage starting from the start codon of the N gene to the stop codon of the large (L) gene (31), were obtained from 36 (GQ20), 34 (DQ20), 23 (GQ25), and 30 (DQ25) specimens (Table 3). The Cp values of the 48 samples ranged from 16 to >35 (Fig. 3). Using the Mann Whitney test, the distribution of Cp values for complete versus incomplete WGS was statistically significantly different (*P* < 0.0001) for each parameter. The 95th percentile successful Cp values for GQ20, DQ20, GQ25, and DQ25 were 31.2, 31.25, 28.68, and 31.4, respectively (Table 3).

**TABLE 3:** Summary of metrics assessed by sequencing of surveillance specimens across the four different ONT pipeline parameters

Metric	Guppy Q20	Dorado Q20	Guppy Q25	Dorado Q25
Depth of coverage threshold	10	10	8	6
95% Successful mean Cp value	31.2	31.25	28.68	31.4
Median depth of coverage (inter-quartile range)	5285 (3469–6635)	1968 (395–5368)	3215 (1771–4913)	1058 (219–3337)
Median sub-consensus nucleotide error rate, % (inter-quartile range)	0.02 (0.00–0.06)	0.00 (0.00–0.06)	0.00 (0.00–0.03)	0.00 (0.00–0.03)
Median sub-consensus deletion rate, % (inter-quartile range)	0.40 (0.19–0.91)	0.53 (0.22–1.27)	0.65 (0.28–1.74)	0.89 (0.35–2.14)
Complete genomes (% of attempted)	36 (75%)	34 (71%)	23 (48%)	30 (63%)
No. of accurate genomes (% of complete)	27 (84.4%)	26 (83.9%)	21 (95.5%)	24 (85.7%)
Reproducibility (% of complete)	37 (97.4%)	33 (91.7%)	27 (96.4%)	31 (91.2%)
Error rate vs Sanger N450	1.4 × 10^−4^	1.4 × 10^−4^	1.0 × 10^−4^	1.6 × 10^−4^
Error rate vs Sanger MF-NCR	6.1 × 10^−5^	6.3 × 10^−5^	8.9 × 10^−5^	7.0 × 10^−5^
Error rate vs Reference WGS	1.2 × 10^−5^	1.2 × 10^−5^	2.9 × 10^−6^	9.1 × 10^−6^

The subsequent analyses were performed with the maximum number of complete genomes that had reference sequences available. This was defined by the GQ20 parameter, which had the most complete genomes (*n* = 36), 32 of which had reference sequences available.

### Sequence metrics of complete WGS from surveillance specimens

**Fig 4:**
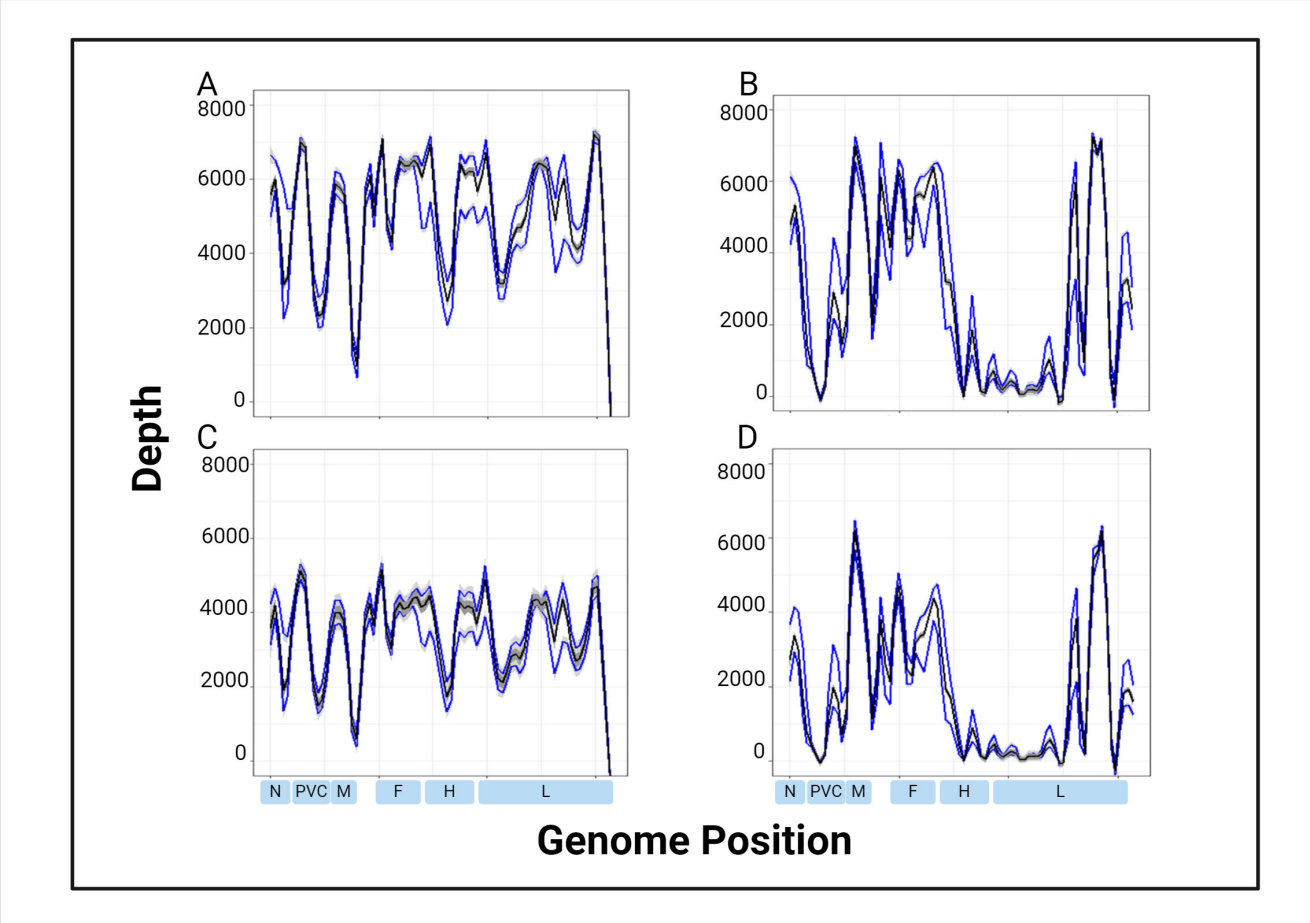
Median coverage depth aggregated across 32 measles genomes. Depth across the genome was determined using QuasiGo of the NanoGO pipeline and plotted by nucleotide position with ggPlot2 in R. Median is plotted in black, and the first and third quartiles are in blue. (A) Guppy Q20, (B) Dorado Q20, (C) Guppy Q25, and (D) Dorado Q25.

The median depth of coverage for the 32 samples used for validation was determined for each of the four parameters (Fig. 4). GQ20 achieved the highest median depth at 5,285, and DQ25 had the lowest median depth at 1,058 (Table 3). Dorado uses duplex basecalling, thus resulting in a lower output of reads. In the Guppy basecalled data, there was a noteworthy drop in coverage near the MF-NCR region. In the Dorado basecalled data, there were other noteworthy drops in coverage in the N to phosphoprotein (P) noncoding region, as well as most of the hemagglutinin (H) and L genes.

**Fig 5:**
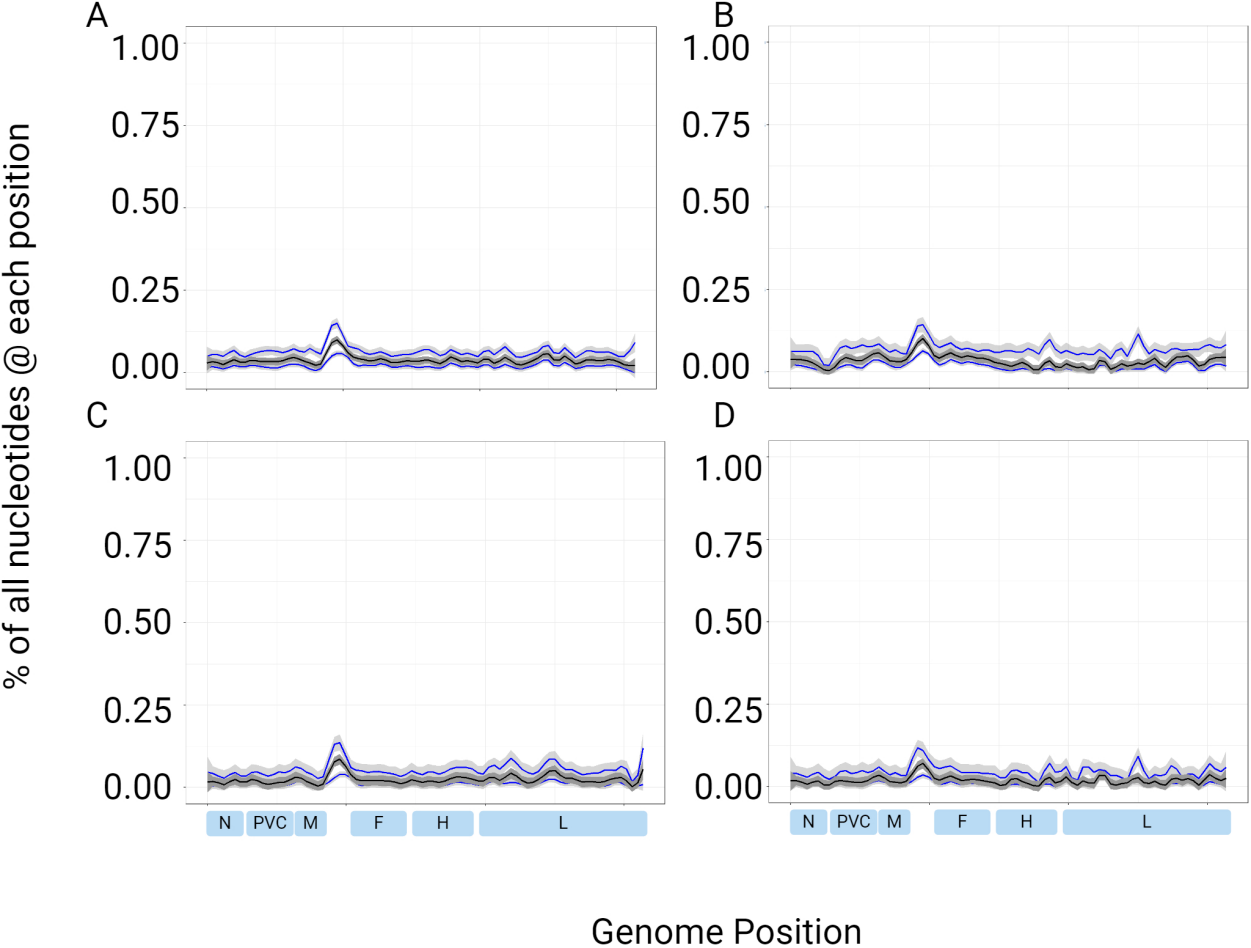
Sub-consensus nucleotide error rates. The count of incorrect nucleotides from the read pileup were quantified and expressed as a percentage of the total number of reads for each position across the genome. Median lines are shown in black; first and third quartiles are shown in blue. (A) Guppy Q20, (B) Dorado Q20, (C) Guppy Q25, and (D) Dorado Q25. The plots were generated in R using ggplot2 and the geom_smooth function.

**Fig 6:**
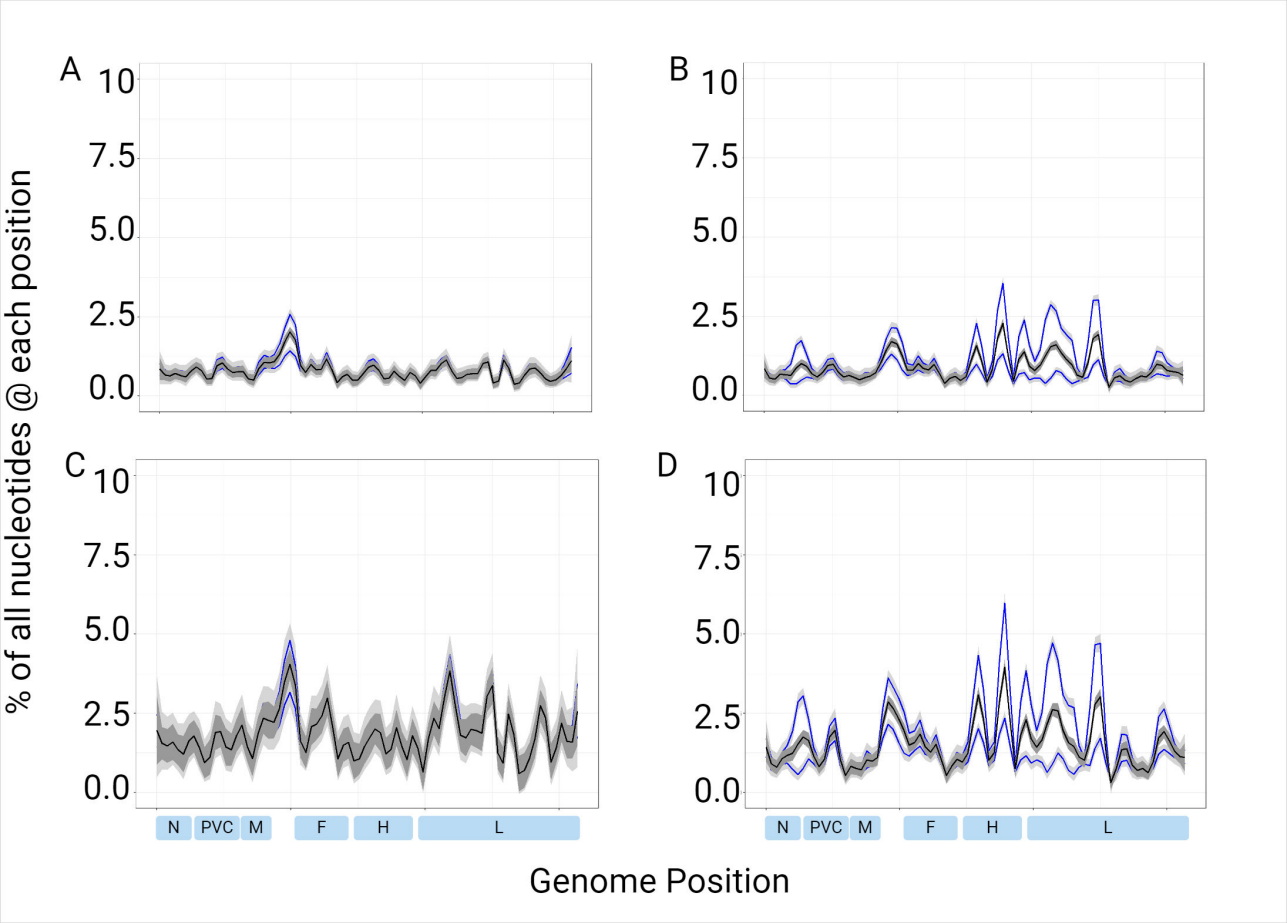
Sub-consensus deletion error rates. The counts of incorrect deletions (gaps in the read pileup) was quantified and expressed as a percentage of the total number of reads for each position across the genome. Median lines are shown in black; first and third quartiles are shown in blue. (A) Guppy Q20, (B) Dorado Q20, (C) Guppy Q25, and (D) Dorado Q25. The plots were generated in R using ggplot2 and the geom_smooth function.

To determine if there were regions of the genome with more sub-consensus level errors than others, we determined the rates of nucleotide errors and deletions (counted as a gap in the read, which can be visualized in the read pileup and is obtained from the QuasiGO file) in the read data across the four parameters. The respective counts at each position in the genome were expressed as percentages of total reads with medians plotted by position (Fig. 5 and 6) and overall medians determined (Table 3). For all four parameters, there appeared to be a trend of higher background read error rates in the MF-NCR, and for DQ20, GQ25, and DQ25, in the H and L genes (Fig. 5). The MF-NCR has many homopolymer stretches and is known to be a difficult region to sequence. The median sub-consensus deletion rates for GQ20, DQ20, GQ25, and DQ25 are 0.40, 0.53, 0.65, and 0.89, respectively (Table 3). The deletion rate was higher for the parameters with a minimum quality score of 25. This is the result of lower quality nucleotides being removed, resulting in more gaps, in addition to any gaps already present in the read data.

### WGS accuracy with surveillance specimens

**Fig 7:**
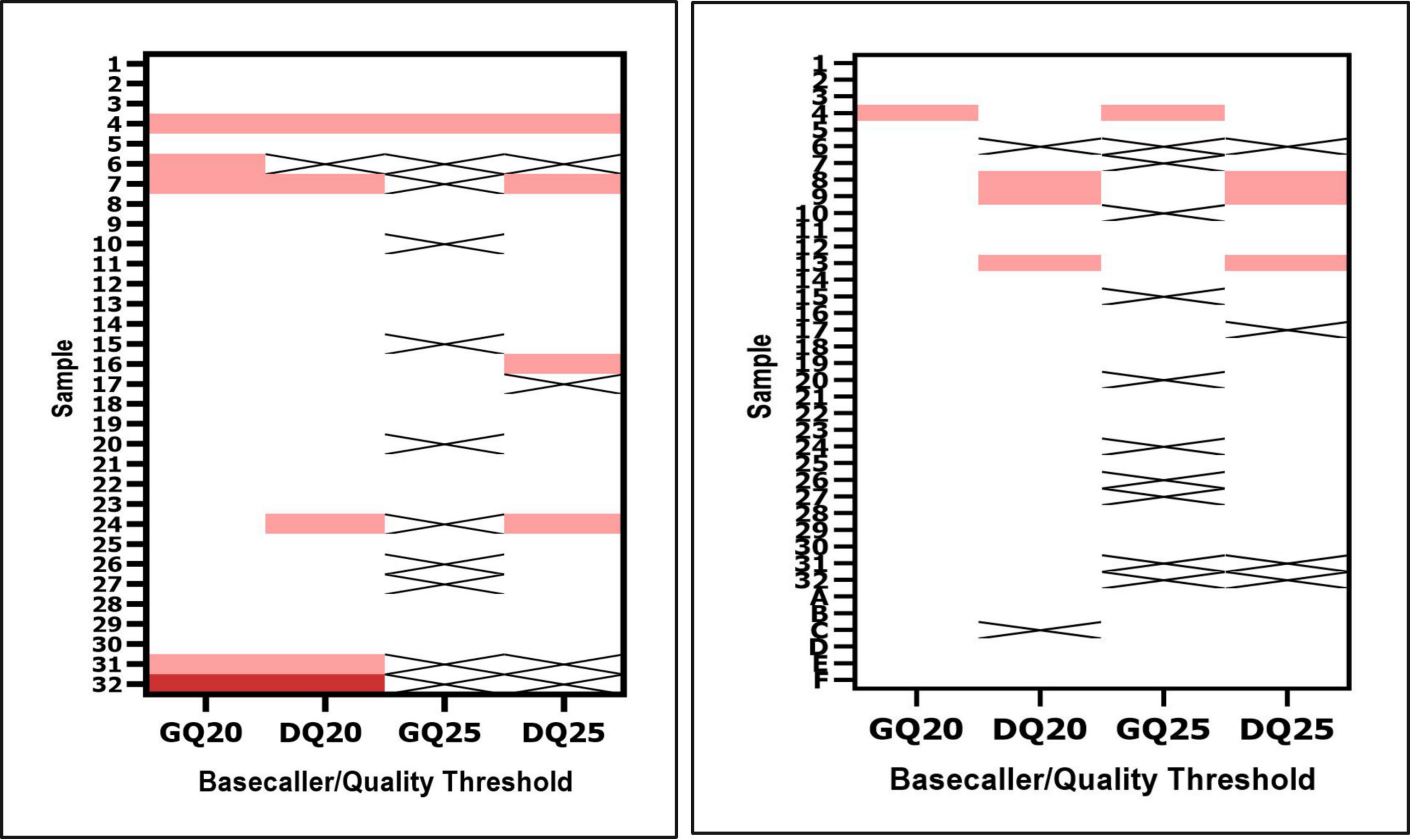
Summary of the accuracy (left) and reproducibility (right) for ONT-generated consensus sequences with four different pipeline parameters. For accuracy, 32 samples were compared with a reference sequence. For reproducibility 32 samples from the same library preparation were run twice on two different flow cells. In addition, a single viral was sequenced six times using different library preparations and flow cells (samples A–F at the bottom of panel B). White values indicate an identical match to the reference sequence, pink values indicate one nucleotide mismatch, red values indicate two nucleotide mismatches, and X’s indicate incomplete genomes. GQ20: Guppy basecaller, quality Phred score 20, DQ20: Dorado basecaller, quality Phred score 20, GQ25: Guppy basecaller, quality Phred score 25, DQ25: Dorado basecaller, quality Phred score 25.

The ONT-generated consensus sequences for the 32 validation samples were compared with a matched reference sequence (Fig. 7, left). The GQ20 pipeline had the greatest number of complete genomes (*n* = 32), 27 (84.4%) of which were identical to the reference sequences. Of the remaining five, four had one mismatch to the reference, and one had two mismatches to the reference, resulting in an error rate of 1.2 × 10^−5^ per nucleotide and 0.19 expected nucleotide errors per MeV WGS (Table 3). For the DQ20 parameter, 26 of 31 complete ONT WGS were identical to the reference (83.9%). Four ONT WGS had one mismatch to the reference, and one had two mismatches for an error rate of 1.2 × 10^−5^ per nucleotide (0.19 expected nucleotide errors per MeV WGS). The pipelines with a quality score of 25 led to fewer complete genomes (22 with Guppy and 28 with Dorado); however, there was higher accuracy for the complete genomes (21, 95.5%, with Guppy and 24, 85.7% with Dorado) (Fig. 7, left). The error rates were 2.9 × 10^−6^ per nucleotide (0.05 expected nucleotide errors per MeV WGS) for GQ25, and 9.1 × 10^−6^ per nucleotide (0.14 expected nucleotide errors per MeV WGS) for DQ25 (Table 3). Based on these data, less than one error in the MeV WGS could be expected using this methodology.

The N450 region and MF-NCR for the 32 ONT-generated sequences were also compared with Sanger-only generated sequences (Table 3). Here, the error rates were generally higher than what had been determined using the reference WGS, with the error rates determined using only the N450 sequence at least 10-fold higher. When extrapolated to the genome, these error rates would result in 0.96–2.18, 0.99–2.25, 1.40–1.58, and 1.10–2.49 nucleotide errors per MeV WGS for the GQ20, DQ20, GQ25, and DQ25 pipelines, respectively.

### WGS reproducibility with surveillance specimens

For the 32 validation samples, the same library preparation was run on two different flow cells to assess sequencing variability (Fig. 7, right). In addition, one isolate was run six times with different library preparations and different flow cells to assess reproducibility for all stages of the protocol resulting in 38 total templates for this analysis. GQ20 had 37 WGS that were identical when repeated (97.4%). There was a single nucleotide mismatch in the replicates (R4964G), with the G an identical match to the reference sequence. DQ20 had 33 WGS that were identical when repeated (91.7%), whereas two were incomplete; three samples had one mismatch in the replicates. GQ25 had 27 WGS that were identical (96.4%) when repeated with 10 incomplete; one sample had one mismatch. DQ25 had 31 that were identical when repeated (91.2%) with four incomplete; three samples each had a single mismatch.

### Selection of pipeline parameters

The outcomes of all analyses were reviewed by parameter (Table 3). GQ20 produced the highest number of complete WGS, had high accuracy, and the best reproducibility. Consensus sequences generated by the GQ20 parameter were used for the subsequent analyses.

### Comparison of ONT GQ20 WGS to reference sequences

For the 32 validation samples with complete ONT WGS (GQ20), there were in total six mutations in five samples: two of those mutations were ambiguous bases, whereas four mutations were nucleotide differences. The genome locations of the mutations were: M gene (one deviation), the MF-NCR (three differences, two of which were mixed reads), the H gene (one difference), and the L gene (one difference) (Fig. S5).

**Fig 8:**
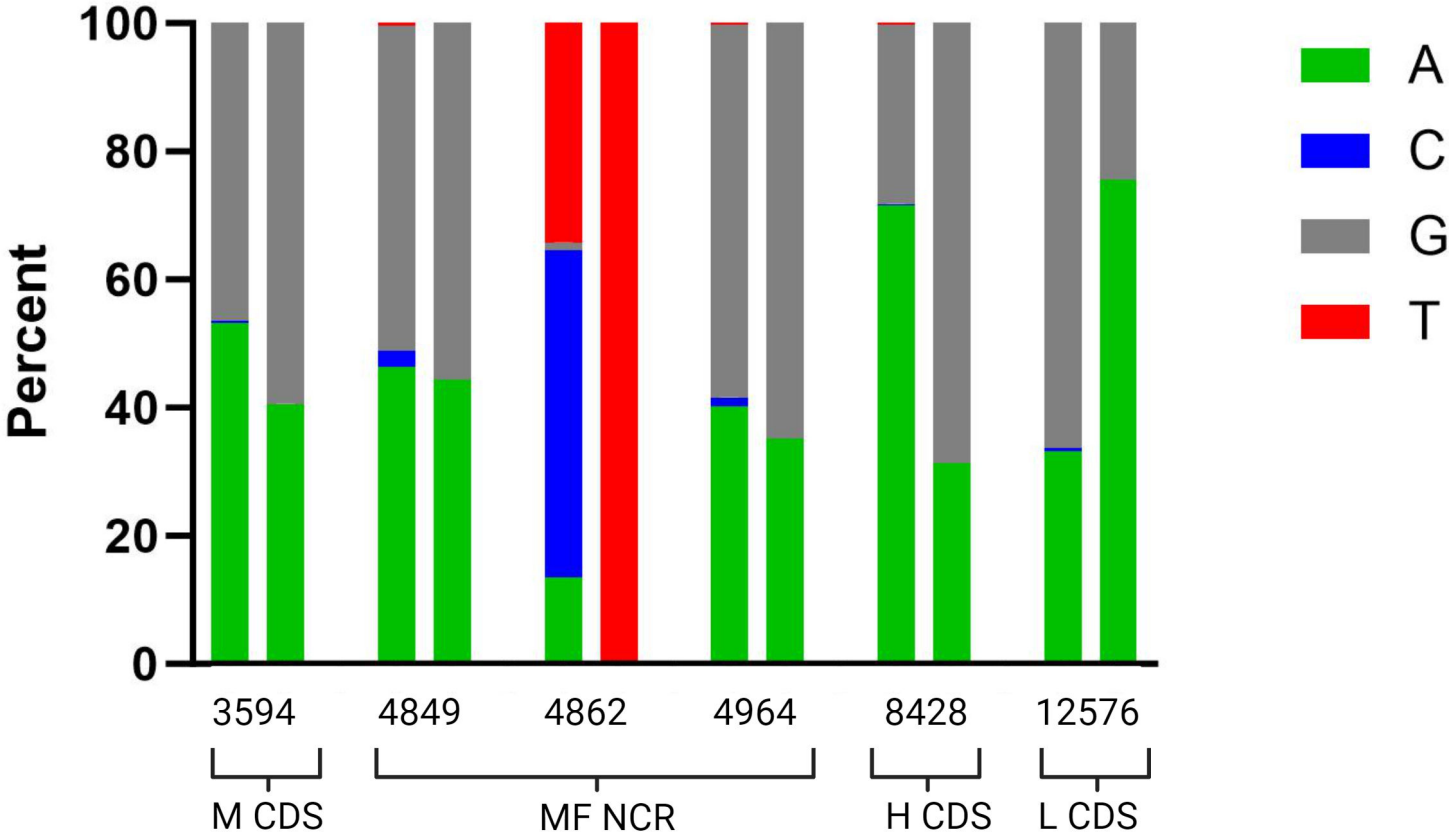
Nucleotide distribution of paired reads for Illumina (left in the pair) and ONT, using the Guppy Q20 pipeline, (right in the pair) by location of the genome for the six positions with consensus sequence level differences. The x-axis indicates the location and region of the genome where the mismatch occurred.

We determined the percentage of each nucleotide present at these sites for both the ONT and Illumina read pileups (Fig. 8). For all six sites, the minimum depth of coverage for Illumina and ONT samples did not go below 423 and 1,570, respectively. The Illumina read pileups indicated a mixed population of reads for all six. For five of these mutations, the ONT results also show mixed populations of nucleotides.

### Generation of WGS, validated by epidemiological data

**Fig 9:**
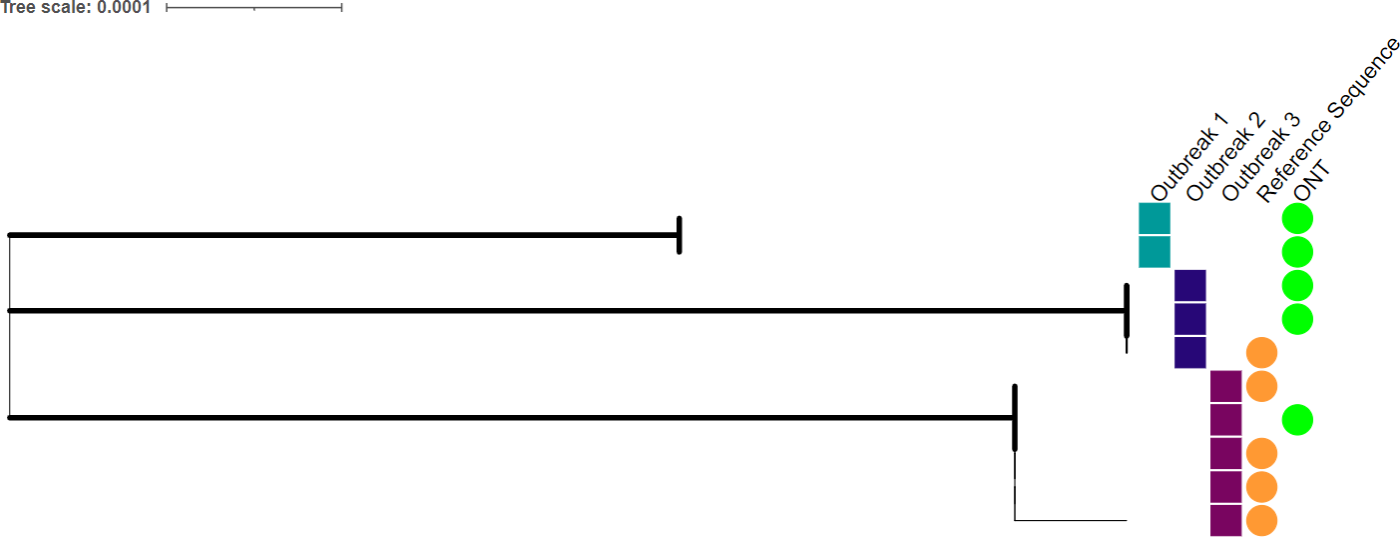
Maximum likelihood phylogenetic tree, of ONT and reference-generated measles WGS with 500 bootstrap replicates, of epidemiologically linked cases. Three different outbreaks are defined by the colored squares and the two sequencing methods are defined by the colored circles.

Finally, we generated sequences from four cases of measles that did not have a paired reference sequence. These cases had epidemiological data linking them to at least one other case, which we leveraged to determine how well the phylogenetic analysis of the ONT-generated WGS fit with the expected linkages (Fig. 9). These four cases were associated with three outbreaks. Outbreak 1 consisted of two cases that were epidemiologically related. Reference sequences for the two cases were not successful. The ONT WGS from the two cases were identical, as expected for linked cases. Outbreak 2 had two cases that were epidemiologically related. There was both a reference sequence and a ONT sequence for one of the cases, whereas the second case had only a ONT WGS. The two WGS generated by ONT were identical to that of the reference sequence. Outbreak 3 consisted of five cases that were all known to be epidemiologically related, and four cases had reference WGS. The WGS for the fifth case was generated by ONT. All five cases clustered together on the phylogenetic tree with the ONT WGS identical to that of three of the reference WGS. One reference WGS had a single nucleotide difference from the other four cases. Thus, all newly generated ONT WGS, without paired reference sequences, were consistent with what was expected based on the epidemiological evidence.

## DISCUSSION

ONT is an inexpensive and convenient platform for WGS, which can be deployed in low-resource, non-specialized laboratories. The reported ONT lower sequencing fidelity (14, 15, 17) could be an obstacle for an application to measles molecular surveillance, where two or three mutations per genome may be enough to identify different chains of transmission. Here, we describe a methodological approach to MeV WGS using ONT, which is accurate enough to be used for measles molecular surveillance.

We assessed Guppy and Dorado basecallers using quality Phred scores of 20 and 25. Using the read data, we determined a minimum coverage threshold for each parameter, which ranged from 6 to 10 and was consistent with the analysis from the downsampling of the reference genome and the 10 read cutoff commonly used for measles WGS generated by Illumina (32). Based on the sequence data from both a dilution series of a quantified reference genome and the real-time RT-PCR Cp distribution of clinical specimens, we found that a Cp value of 31 (corresponding to approximately 100 copies/µL) or less could be considered a predictor for the generation of accurate and complete WGS.

GQ20 had the highest number of successful samples (*n* = 36) and the highest depth of coverage. We found that the sub-consensus nucleotide error rates were exceptionally low and sub-consensus deletion rates (captured as gaps in the read data compared with the expected sequence) were higher. As our quality threshold increased from a Phred score of 20–25, the deletion rate increased. Deletions cannot be screened out while the filtering of lower quality reads, this results in a decrease of reads, and thus, an overall increase in the relative abundance of deletions. Deletions for the Q20 threshold tended to be in the MF-NCR, a region with long stretches of homopolymers, a known weakness of the ONT platform, which manifests as insertions and deletions (19). For this reason, reference-based assemblers are recommended rather than *de novo* assembly. GQ20 had the best reproducibility out of the four parameters, with only one mismatch in one sample between sequencing runs. This mismatch that occurred was an R to a G, and this change would have made the sequence an identical match to the reference sequence. We chose the GQ20 parameter because it had the best balance of depth of coverage and sub-consensus data quality (deletions and errors), which contributed to higher sensitivity (as measured by the Cp values of the successful specimens and completion rate), reproducibility, and accuracy.

When compared with the reference sequences, the ONT tiled amplicon methodology developed here combined with the GQ20 pipeline, would result in less than one predicted nucleotide error for the entire MeV WGS. A report from 2022 comparing ONT with Illumina for gram-negative bacteria found a nucleotide error rate of 2 × 10^−5^ (23). The error rate identified here for measles is similar at 1.2 × 10^−5^. Using the Sanger sequences of the N450 and MF-NCR regions, the error rates found for the ONT were 1.4 × 10^−4^ and 6.1 × 10^−5^, respectively, which is slightly higher than the rate previously determined using an expanded set of specimens sequenced by Illumina (8.7 × 10^−5^ and 1.6 × 10^−5^, respectively) (16). However, this would result in only 1–2 predicted errors in nucleotide assignment over the MeV WGS, of 15,678 nucleotides in length, with this ONT method, whereas 0–1 are predicted for Illumina.

In comparison to the reference sequences, six differences were identified in the consensus sequence generated by the ONT method. Inspection of the read data for both the Illumina and ONT platforms revealed that a mixture of nucleotides was present for all six sites in the Illumina data, and ONT showed a mixture of nucleotides for five of the sites. The standard practice in the global measles and rubella laboratory network is to achieve exact, unambiguous, nucleotide assignments for measles sequences; ambiguous nucleotides are not accepted in the measles sequence database (MeaNS). Thus, the reference sequences used in this study were defined by a majority consensus requiring only 51% of reads, which masked the presence of any nucleotides of mixed populations such as these. As a result, these sites were identified as errors in the ONT consensus sequence, but upon closer review, it appears that it is an accurate representation of the samples themselves, as corroborated by the Illumina data. The issue then is the lack of tolerance for mixed populations in measles sequence data that perhaps should be reviewed with respect to WGS data. Finally, we explored inserting the ONT and Illumina-generated sequences into outbreaks together with known epidemiological links. The results conclude that the ONT-generated WGS are accurate and can be used to confirm related cases and show transmission.

This study has some limitations. First, the methodology would be enhanced by the addition of the WGS for the other circulating MeV genotype, B3. Initially, we began this project as a proof of concept to understand and attempt to employ MeV WGS with ONT, and hence, the analysis was restricted to genotype D8, for which we had many specimens with pre-determined WGS (the reference sequences) available. Future work will include expansion of the assay to include genotype B3. Another limitation is the bench protocol consists of six different primer pools, which is not well suited for the sequencing of many specimens at a time. However, we found that it was necessary to have additional pools for adequate coverage of the large non-coding region, the MF-NCR, which is in low abundance, riddled with many homopolymer stretches and secondary structures. In addition, we did not perform any testing on the Flongle flow cell, which is an economical option that allows for very few and on-demand samples to be processed. At the time of conducting this study, the R10 version of the Flongle flow cell was not yet available. Finally, we note that ONT is constantly changing and improving its technology. This evaluation represents data generated with the stated ONT versions that were available at the time.

In conclusion, we developed and validated an ONT-tiled amplicon bench protocol and bioinformatics pipeline for measles WGS that has been compared with reference WGS generated by Illumina and Sanger platforms. We found that this protocol accurately and reproducibly generates MeV WGS that are comparable with those generated by Illumina; however, we note that it requires a more in-depth bioinformatics pipeline. We feel that with some additional effort to enhance the genotype suitability of the tiled amplicon approach, this ONT methodology, which is more affordable and deployable than other methods, has the potential to increase the speed in which measles WGS are generated, in elimination settings, facilitating outbreak analysis and response, and expand capacity to regions for which there are limited sequence data.

## Data Availability

All raw reads for data basecalled with Guppy and Dorado have been submitted to SRA (Sequence Read Archive), under Bioproject PRJNA1174053. Reference sequence data has been published under BioProject PRJNA1017431 with the consensus sequences published on GenBank.

## SUPPLEMENTAL MATERIAL

Supplemental figures jcm.01456-24-s0001.docx Figures S1 to S5.

ASM does not own the copyrights to Supplemental Material that may be linked to, or accessed through, an article. The authors have granted ASM a non-exclusive, world-wide license to publish the Supplemental Material files. Please contact the corresponding author directly for reuse.

## References

[1] Tulchinsky TH. 2018. Maurice Hilleman: creator of vaccines that changed the world. Case Studies in Public Health 2018:443–470. doi:10.1016/B978-0-12-804571-8.00003-2

[2] Minta AA, Ferrari M, Antoni S, Portnoy A, Sbarra A, Lambert B, Hatcher C, Hsu CH, Ho LL, Steulet C, Gacic-Dobo M, Rota PA, Mulders MN, Bose AS, Caro WP, O’Connor P, Crowcroft NS. 2023. Progress toward measles elimination - worldwide, 2000-2022. MMWR Morb Mortal Wkly Rep 72:1262–1268. doi:10.15585/mmwr.mm7246a3 37971951 PMC10684353

[3] Global measles threat continues to grow another year passes with millions of children unvaccinated. 2023. Joint News Release. Available from: https://www.who.int/news/item/16-11-2023-global-measles-threat-continues-to-grow-as-another-year-passes-with-millions-of-children-unvaccinated. Retrieved 16 Nov 2023.

[4] Statement from the Chief Public Health Officer of Canada Update on Measles and Risk to Canadians. Accessed 1 April 2024

[5] CDC Measles cases and outbreaks. Accessed 1 April 2024

[6] Regular measles statistics updates issued by the UK Health Security Agency (UKHSA). 2024. Available from: https://www.gov.uk/

[7] European centre for disease prevention and control weekly Communicable Disease Threats report. 2024-WCP-0018 Draft.Docx (Europa.Eu). Accessed 1 April 2024

[8] Government of Canada. 2024. Measles Prevention and Risks. Available from: https://www.canada.ca/en/public-health/services/diseases/measles/prevention-risks.html

[9] World Health Organization = Organisation mondiale de la Santé. 2022. Update: circulation of active genotypes of measles virus and recommendations for use of sequence analysis to monitor viral transmission – Mise à jour sur la circulation des génotypes actifs du virus rougeoleux et recommandations d’utilisation de l’analyse de séquence pour surveiller la transmission virale. Weekly Epidemiological Record = Relevé épidémiologique hebdomadaire 97:485–492. https://iris.who.int/handle/10665/363332

[10] Rota JS, Heath JL, Rota PA, King GE, Celma ML, Carabaña J, Fernandez-Muñoz R, Brown D, Jin L, Bellini WJ. 1996. Molecular epidemiology of measles virus: identification of pathways of transmission and implications for measles elimination. J Infect Dis 173:32–37. doi:10.1093/infdis/173.1.32 8537679

[11] Rota PA, Brown KE, Hübschen JM, Muller CP, Icenogle J, Chen M-H, Bankamp B, Kessler JR, Brown DW, Bellini WJ, Featherstone D. 2011. Improving global virologic surveillance for measles and rubella. J Infect Dis 204:S506–S513. doi:10.1093/infdis/jir117 21666207

[12] Bankamp B, Kim G, Hart D, Beck A, Ben Mamou M, Penedos A, Zhang Y, Evans R, Rota PA. 2024. Global update on measles molecular epidemiology. Vaccines (Basel) 12:810. doi:10.3390/vaccines12070810 39066448 PMC11281501

[13] Crossley BM, Bai J, Glaser A, Maes R, Porter E, Killian ML, Clement T, Toohey-Kurth K. 2020. Guidelines for sanger sequencing and molecular assay monitoring. J VET Diagn Invest 32:767–775. doi:10.1177/1040638720905833 32070230 PMC7649556

[14] Slatko BE, Gardner AF, Ausubel FM. 2018. Overview of next-generation sequencing technologies. Curr Protoc Mol Biol 122:e59. doi:10.1002/cpmb.59 29851291 PMC6020069

[15] Zhang T, Li H, Ma S, Cao J, Liao H, Huang Q, Chen W. 2023. The newest Oxford Nanopore R10.4.1 full-length 16S rRNA sequencing enables the accurate resolution of species-level microbial community profiling. Appl Environ Microbiol 89:e0060523. doi:10.1128/aem.00605-23 37800969 PMC10617388

[16] Hiebert J, Zubach V, Schulz H, Severini A. 2024. Genomic tools for post-elimination measles molecular epidemiology using Canadian surveillance data from 2018-2020. Front Microbiol 15:1475144. doi:10.3389/fmicb.2024.1475144 39629208 PMC11611582

[17] MacKenzie M, Argyropoulos C. 2023. An introduction to nanopore sequencing: past, present, and future considerations. Micromachines (Basel) 14:459. doi:10.3390/mi14020459 36838159 PMC9966803

[18] 2024. Oxford Nanopore Technologies. Available from: https://nanoporetech.com/platform/accuracy

[19] Sereika M, Kirkegaard RH, Karst SM, Michaelsen TY, Sørensen EA, Wollenberg RD, Albertsen M. 2022. Oxford Nanopore R10.4 long-read sequencing enables the generation of near-finished bacterial genomes from pure cultures and metagenomes without short-read or reference polishing. Nat Methods 19:823–826. doi:10.1038/s41592-022-01539-7 35789207 PMC9262707

[20] Wang Y, Zhao Y, Bollas A, Wang Y, Au KF. 2021. Nanopore sequencing technology, bioinformatics and applications. Nat Biotechnol 39:1348–1365. doi:10.1038/s41587-021-01108-x 34750572 PMC8988251

[21] Bull RA, Adikari TN, Ferguson JM, Hammond JM, Stevanovski I, Beukers AG, Naing Z, Yeang M, Verich A, Gamaarachchi H, Kim KW, Luciani F, Stelzer-Braid S, Eden J-S, Rawlinson WD, van Hal SJ, Deveson IW. 2020. Analytical validity of nanopore sequencing for rapid SARS-CoV-2 genome analysis. Nat Commun 11:6272. doi:10.1038/s41467-020-20075-6 33298935 PMC7726558

[22] McNaughton AL, Roberts HE, Bonsall D, de Cesare M, Mokaya J, Lumley SF, Golubchik T, Piazza P, Martin JB, de Lara C, Brown A, Ansari MA, Bowden R, Barnes E, Matthews PC. 2019. Illumina and Nanopore methods for whole genome sequencing of hepatitis B virus (HBV). Sci Rep 9:7081. doi:10.1038/s41598-019-43524-9 31068626 PMC6506499

[23] Lerminiaux N, Fakharuddin K, Mulvey MR, Mataseje L. 2024. Do we still need Illumina sequencing data? Evaluating Oxford Nanopore Technologies R10.4.1 flow cells and the rapid v14 library prep kit for Gram negative bacteria whole genome assemblies. Can J Microbiol 70:178–189. doi:10.1139/cjm-2023-0175 38354391

[24] Quick J, Grubaugh ND, Pullan ST, Claro IM, Smith AD, Gangavarapu K, Oliveira G, Robles-Sikisaka R, Rogers TF, Beutler NA, et al.. 2017. Multiplex PCR method for MinION and Illumina sequencing of Zika and other virus genomes directly from clinical samples. Nat Protoc 12:1261–1276. doi:10.1038/nprot.2017.066 28538739 PMC5902022

[25] Rozen S, Skaletsky H, Primer 3 software. 2000. Primer3 on the WWW for general users and for biologist programmers. Methods Mol Biol 132:365–386. doi:10.1385/1-59259-192-2:365 10547847

[26] Ye J, Coulouris G, Zaretskaya I, Cutcutache I, Rozen S, Madden TL. 2012. Primer-BLAST: a tool to design target-specific primers for polymerase chain reaction. BMC Bioinformatics 13:134. doi:10.1186/1471-2105-13-134 22708584 PMC3412702

[27] Canadian Institutes of Health Research, Natural Sciences and Engineering Research Council of Canada, and Social Sciences and Humanities Research Council of Canada. 2022. TriCouncil Policy Statement: Ethical Conduct for Research Involving Humans

[28] Zubach V, Severini A, Hiebert J. 2022. Development of a rapid, internally controlled, two target, real-time RT-PCR for detection of measles virus. J Virol Methods 299:114349. doi:10.1016/j.jviromet.2021.114349 34740707

[29] Kumar S, Stecher G, Li M, Knyaz C, Tamura K. 2018. MEGA X: molecular evolutionary genetics analysis across computing platforms. Mol Biol Evol 35:1547–1549. doi:10.1093/molbev/msy096 29722887 PMC5967553

[30] Letunic I, Bork P. 2021. Interactive Tree Of Life (iTOL) v5: an online tool for phylogenetic tree display and annotation. Nucleic Acids Res 49:W293–W296. doi:10.1093/nar/gkab301 33885785 PMC8265157

[31] Global Measles and Rubella Laboratory Network meeting. 2017. Releve Epidemiologique Hebdomadaire. Vol. 93. 16300026

[32] Schulz H, Hiebert J, Frost J, McLachlan E, Severini A. 2022. Optimisation of methodology for whole genome sequencing of measles virus directly from patient specimens. J Virol Methods 299:114348. doi:10.1016/j.jviromet.2021.114348 34728271

