# Supplementary material for: Development and evaluation of a next-generation sequencing methodology for measles virus using Oxford Nanopore Technology
Source: J Clin Microbiol. 2025 Feb 4;63(3):e01456-24. doi: 10.1128/jcm.01456-24 (PMC11898632; doi:10.1128/jcm.01456-24)
Supplement: Supplemental figures — Figures S1 to S5. [file jcm.01456-24-s0001.docx]

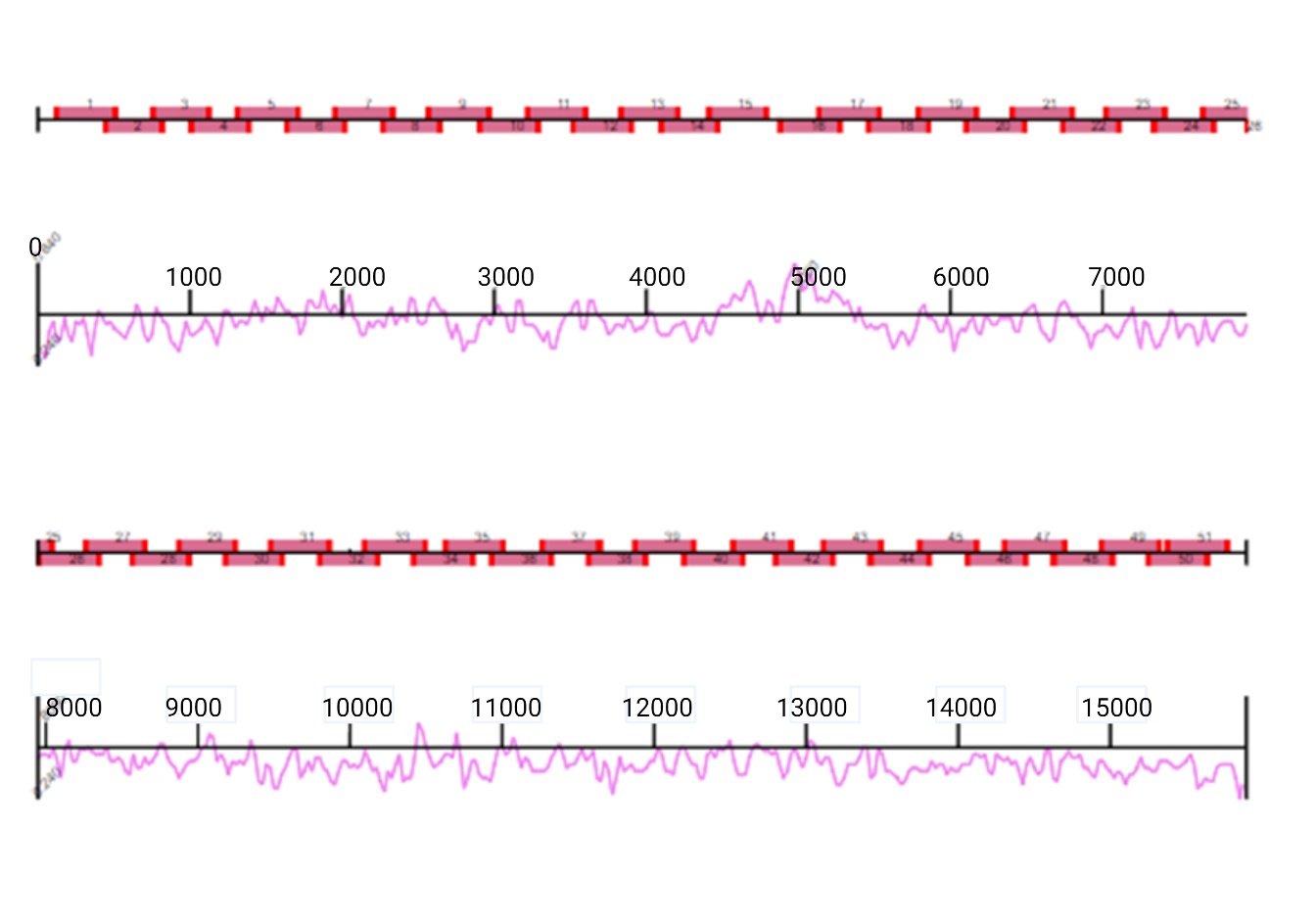
Supplementary Figure 1. Map showing the location of primers designed using Primal Scheme producing 400bp amplicons across the MeV genome. MH356245 was used as a reference genome. The red rectangular boxes represent the amplicons, the jagged purple lines represent the GC content across the MeV genome.


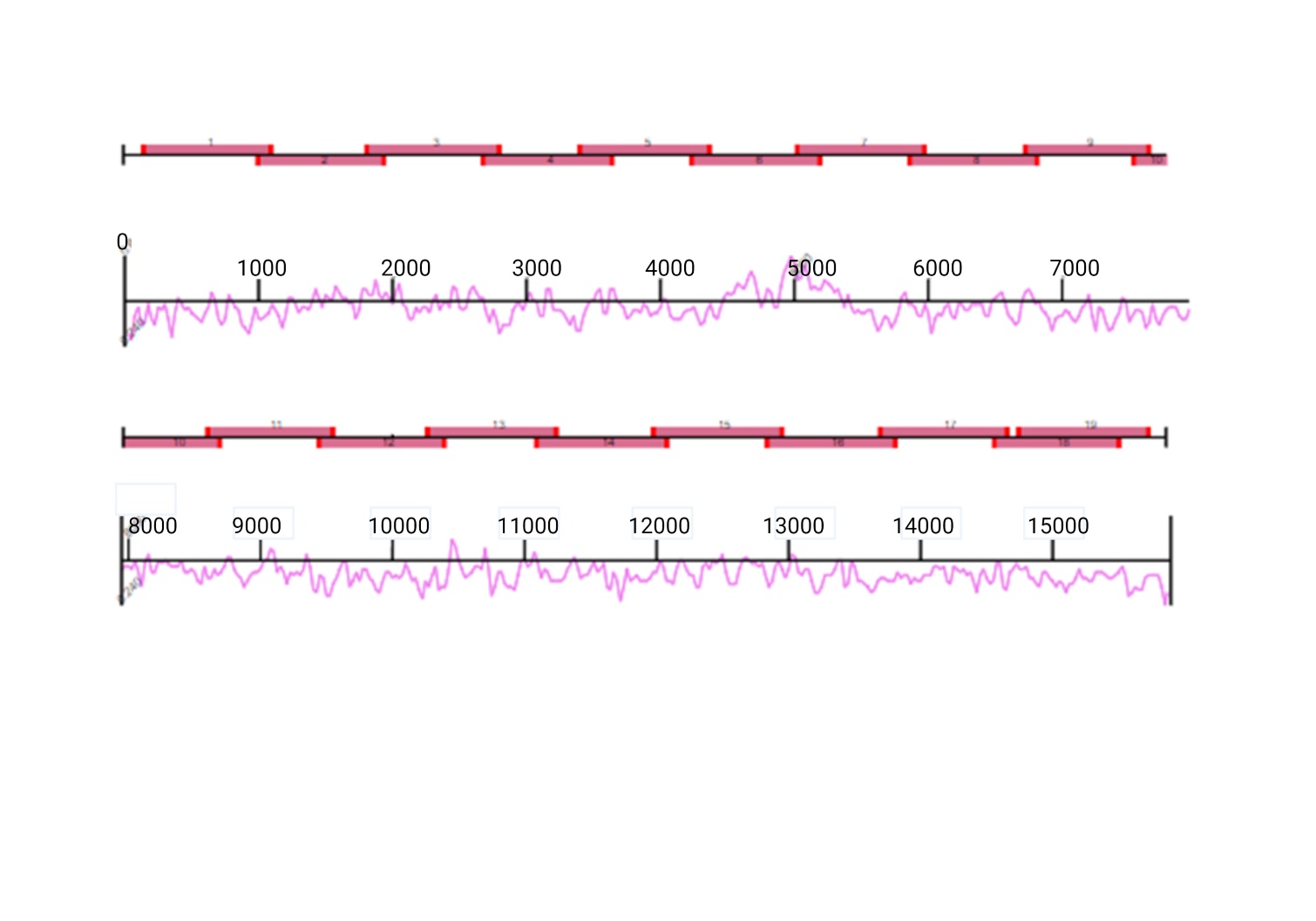


Supplementary Figure 2. Map showing the location of primers designed using Primal Scheme producing 1000bp amplicons across the MeV genome. MH356245 was used as a reference genome. The red rectangular boxes represent the amplicons, the jagged purple lines represent the GC content across the MeV genome.


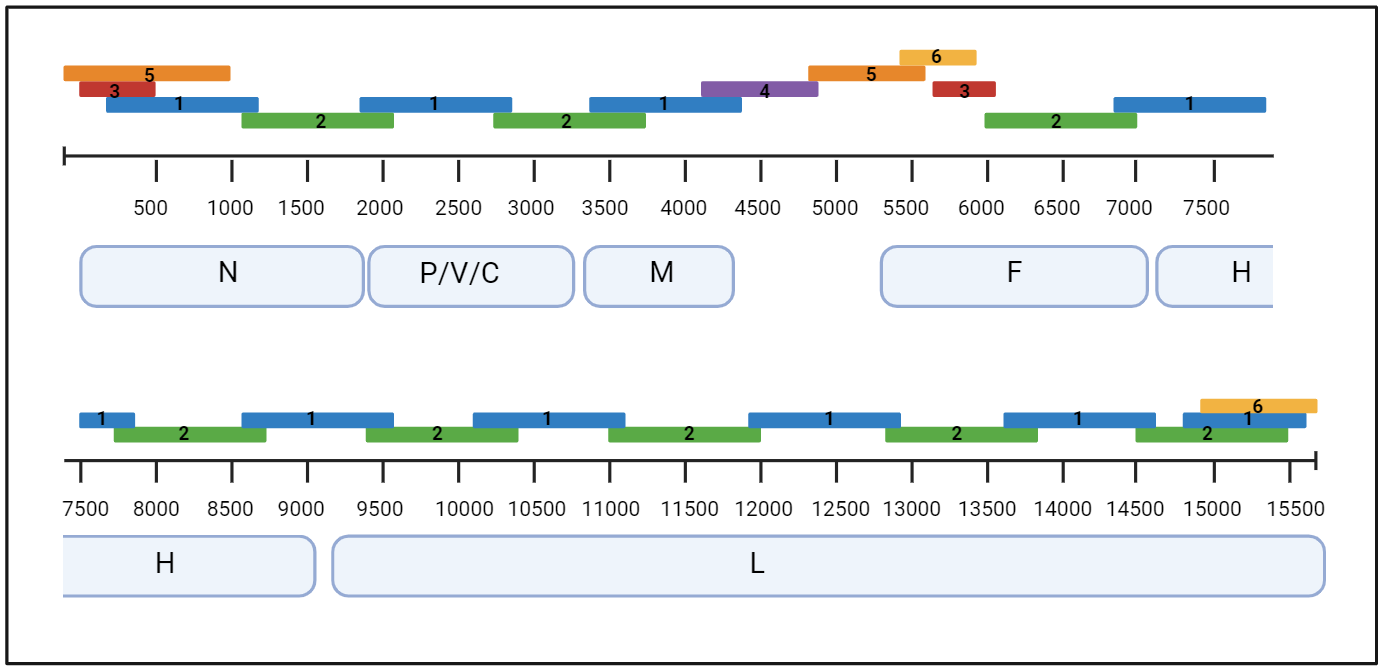


Supplemental Figure 3. Displays the location of the primers and their pools across the measles genome. Primer pools 1 and 2 (blue and green) were developed by primal scheme. Pools 3, 4, 5 and 6 (red, purple, yellow and orange) were supplemental primers developed in-house. Figure created using Biorender.


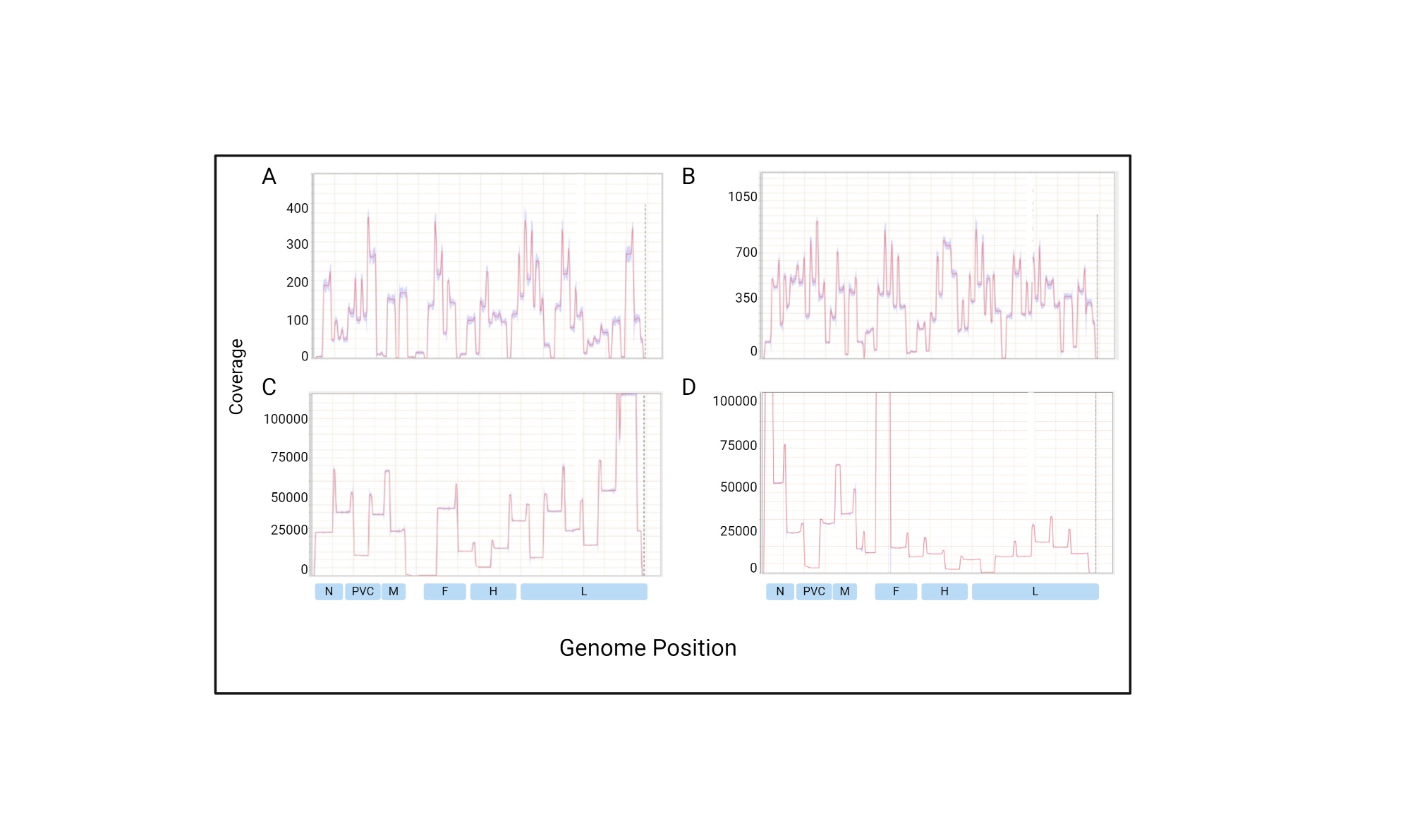
Supplemental Figure 4. Shows the evolution of the bench protocol for our method. Panel A is a library preparation using the 400bp tiled amplicons. Panel B is a library preparation using 400bp amplicons with the addition of betaine at the primer annealing step. Panel C is a library preparation using 1000bp amplicons and with the addition of betaine at the primer annealing step. Panel D is a library preparation using 1000bp amplicons, the addition of betaine at the primer annealing step and supplementary primers. Note the scale of each chart is different. Figure was created using Biorender.


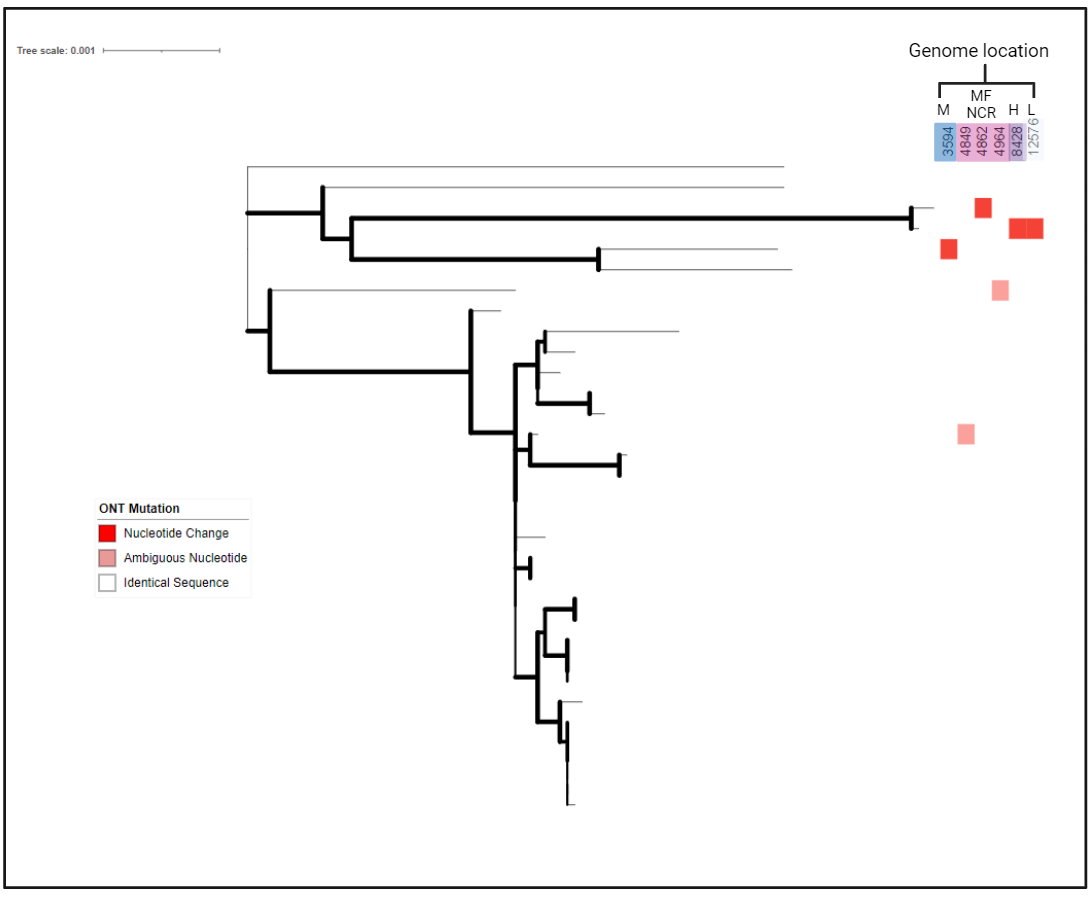


Supplemental Figure 5. Maximum likelihood phylogenetic tree using bootstrapping of 32 ONT generated WGS using GQ20 parameters. Heat map indicates which sample had a mismatch and where in the genome the mismatch occurred. White boxes indicate identical sequences to the gold standard, light red indicate ambiguous bases and red indicate a nucleotide mismatch.
